# Sensory impairment and cognitive decline among older adults: An analysis of mediation and moderation effects of loneliness

**DOI:** 10.3389/fnins.2022.1092297

**Published:** 2023-01-09

**Authors:** Shaoqing Ge, Wei Pan, Bei Wu, Brenda L. Plassman, XinQi Dong, Eleanor S. McConnell

**Affiliations:** ^1^Department of Biobehavioral Nursing and Health Informatics, University of Washington School of Nursing, Seattle, WA, United States; ^2^Duke University School of Nursing, Durham, NC, United States; ^3^Department of Population Health Sciences, Duke University School of Medicine, Durham, NC, United States; ^4^New York University Rory Meyers College of Nursing, New York, NY, United States; ^5^Department of Psychiatry and Neurology, Duke University School of Medicine, Durham, NC, United States; ^6^Rutgers University Institute for Health, Health Care Policy and Aging Research, New Brunswick, NJ, United States; ^7^Geriatric Research Education and Clinical Center (GRECC), Durham Department of Veterans Affairs (VA) Healthcare System, Durham, NC, United States

**Keywords:** sensory impairment, cognitive aging, Alzheimer’s disease, psychosocial, risk factors

## Abstract

**Background:**

Multiple studies have reported that hearing and vision impairment are linked to cognitive decline. Yet little is known about factors that may influence the association between sensory impairment and cognitive decline. This study examined if loneliness mediates or moderates the impact of sensory impairment on cognitive decline as individuals age.

**Methods:**

This was a longitudinal study using data from the Health and Retirement Study (HRS) and The Aging, Demographics, and Memory Study (ADAMS) (*N* = 243). We used one timepoint of hearing and vision (ADAMS 2006–2008), one timepoint of loneliness (HRS 2006–2008), and five waves of cognition (HRS 2006–2014). Hearing impairment was defined by an inability to hear pure-tone stimuli of 25 dB at frequencies between 0.5 and 4.0 kHz in either ear. Visual impairment was defined as having corrected binocular vision worse than 20/40. Longitudinal parallel-process (LPP) analysis was conducted at a significance level of α = 0.05 (one-tailed).

**Results:**

Loneliness moderated but did not mediate the association between visual impairment and the rate of cognitive decline (standardized β =−0.108, *p* < 0.05). No moderation or mediation effect of loneliness was found for the association between hearing impairment and cognitive decline. Both vision and hearing impairment were significantly associated with increased severity of loneliness.

**Conclusion:**

Visual impairment combined with an elevated level of loneliness may produce a more synergistic, deleterious impact on older adults’ cognitive function than visual impairment alone. This study highlights the importance of promoting a healthy social and psychological status for older adults with sensory impairment.

## Introduction

Hearing impairment and visual impairment are highly prevalent among older adults (Lin et al., 2011c; Varma et al., 2016) and have been found to be independently associated with cognitive decline (Reyes-Ortiz et al., 2005; Lin et al., 2011b,^2013^; Deal et al., 2016; Zheng et al., 2018; Ge et al., 2021). Understanding the pathways connecting sensory impairment and cognitive function has been acknowledged as a priority for future research (Whitson et al., 2018). Loneliness, or perceived social isolation (Cacioppo et al., 2011), has been hypothesized to be both a potential moderator (Hämäläinen et al., 2019) and mediator (Lin et al., 2011a,b; Fulton et al., 2015) between sensory impairment and cognitive function. A better understanding of these relationships would support the development of targeted interventions to prevent cognitive decline among the growing population of older adults.

Theoretically, loneliness has been hypothesized to partially explain the association between sensory impairment and cognitive function (Fulton et al., 2015). However, empirical evidence for this mediational relationship is scarce and inconsistent; the prior studies are also methodologically limited by the use of self-reported hearing impairment (Maharani et al., 2019) or being cross-sectional (Hämäläinen et al., 2019). Longitudinal studies exploring the potential mediation effect of loneliness in the relationship of either hearing or vision impairment with cognitive decline using objectively measured sensory function are warranted.

In addition to the potential for loneliness to mediate the effects of sensory impairment on cognitive decline, prior studies have also suggested that loneliness may moderate this relationship (Hämäläinen et al., 2019). One might expect lonely individuals to demonstrate a stronger association between sensory impairment and cognitive decline given previous research showing that lonely individuals tend to view sensory impairment as more stressful (Hawkley and Cacioppo, 2003). Loneliness is an important psychosocial factor for older adults and has been found to moderate the relationship between life stressors and health outcomes (Norman et al., 2011; Doane and Thurston, 2014; Zeligman et al., 2017). However, whether loneliness moderates the association between sensory impairment and cognitive decline has not been explored. Previous studies have found older adults who have sensory impairment were more likely to feel lonely (Nachtegaal et al., 2009; Mick et al., 2018), and both sensory impairment and loneliness have been found to be risk factors for cognitive decline (Livingston et al., 2017). Therefore, sensory impairment and loneliness may reinforce each other and produce synergistic, deleterious effects on cognitive function.

The purpose of this study was to better understand the inter-relationships among sensory impairment, changes in cognitive function, and loneliness. Specifically, we aimed to examine (a) if loneliness mediates the association between sensory impairment (i.e., hearing or vision impairment) and cognitive decline over time; and (b) if loneliness moderates the association between sensory impairment (i.e., hearing or vision impairment) and cognitive decline over time. We hypothesized that sensory impairment is associated with cognitive decline via the pathway of an elevated level of loneliness (mediation). We also hypothesized that older adults with both sensory impairment and higher-level of loneliness have worse cognition or faster rates of cognitive decline than those with sensory impairment alone (moderation).

## Methods

### Parent study overview and data source

This was a longitudinal study using data from the Health and Retirement Study (HRS) and its supplement: The Aging, Demographics, and Memory Study (ADAMS) (Plassman et al., 2007). The ongoing Health and Retirement Study (HRS) is a population-based, nationally representative epidemiological survey of U.S. adults aged 51 years and older. Participants were interviewed every 2 years since 1992. The Aging, Demographics, and Memory Study (ADAMS) is an HRS substudy of dementia among older adults aged 70 or older (grant number NIA U01AG009740) (Plassman et al., 2007). Four waves of data collection (waves A, B, C, and D) occurred during Aug 2001–Dec 2003, Nov 2002–Mar 2005, Jun 2006–May 2008, and Jan 2008–Dec 2009. After Wave A, participants without dementia at any given wave were targeted for the next wave of data collection.

**FIGURE 1:**
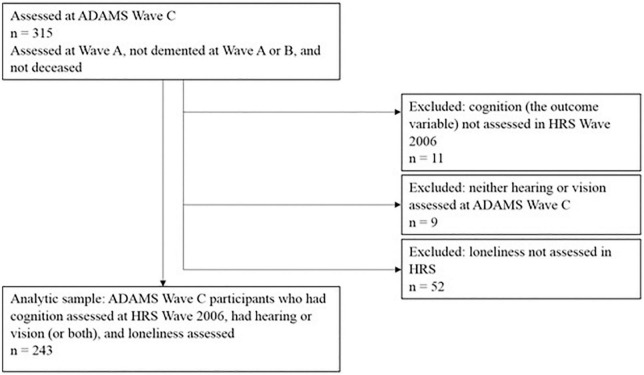
Flow chart for deriving the analytical sample from Aging, Demographics, and Memory Study (ADAMS) Wave C.

Data from the HRS and ADAMS were merged using household and participant IDs. Hearing and vision were objectively measured in ADAMS Wave C (*N* = 315). For this study, we used one wave of hearing and vision data from ADAMS Wave C (Jun 2006–May 2008), one wave of loneliness data from HRS (collected between 2006 and 2008), and five waves of cognitive function data measured in HRS in 2006–2014. Figure 1 demonstrates how we have derived the analytic sample in this study from ADAMS Wave C. Among the 315 older adults with measured sensory status in ADAMS Wave C, 243 had cognitive function and loneliness data from HRS, providing a sample of 243 for the current study. The study was approved by the Duke University IRB.

### Measures

#### Cognitive function

Cognitive function was measured either via telephone or in-person for those aged 65 and older at every wave (every 2 years) of HRS using the HRS Telephone Interview for Cognitive Status (TICS) (Ofstedal et al., 2005). No items of the HRS TICS relied on visual presentation because the measure was designed to be administered over the telephone. Previous research has shown no measurable differences in cognitive performance based on the mode of the test administration in HRS (Herzog and Rodgers, 1999). The HRS TICS included immediate and delayed free-recall (range 0–20), serial 7 subtraction (range 0–5), counting backward tests (range 0–2), orientation to time (range 0–4), object naming (range 0–2), and president/vice president naming (range 0–2) (Brandt et al., 1988). A total cognitive function score was calculated by summing the score for all items (range: 0–35) with a higher score indicating better cognitive function.

#### Hearing impairment

Hearing impairment was measured by administering the Pure Tone Thresholds test (Sensitive Health Data, 2013). Pure tone stimuli were presented at 0.5, 1, 2, and 4 kHz at both 25 and 40 dB to the right and the left ear individually. The response was documented “yes” for the frequency and decibel-level combination if a participant reported being able to hear the 25 or 40 dB stimulus at a specific Hz level, otherwise was “no.” Criteria for hearing impairment were defined based on the American Speech-Language-Hearing Association guidelines and other studies (Clark, 1981; Lin et al., 2011c). We categorized participants as (a) having normal hearing if the better ear could hear 25 dB for at least some of the frequencies, and (b) having hearing impairment if the better ear could not hear 25 dB at any frequencies.

#### Visual impairment

Visual impairment was measured by using a Snellen chart (Sensitive Health Data, 2013). Visual impairment was defined as the best-corrected binocular vision being worse than 20/40. This cut-point has been used in other studies of visual impairment, including large-scale population-based surveys (West et al., 1997; Lin et al., 2004; Reyes-Ortiz et al., 2005). This cut-point has also been used in screening for issuing driver’s license (West et al., 1997).

#### Loneliness

Loneliness was measured by the 3-item UCLA Loneliness Scale in the HRS self-administered leave-behind psychosocial and lifestyle questionnaire (Smith et al., 2017). Participants were asked “How much of the time do you feel you lack companionship?”, “How much of the time do you feel left out?”, and “How much of the time do you feel isolated from others?” The response options ranged from 1 (often) to 3 (hardly ever or never). Loneliness was reverse coded and was calculated as the average score of the three items, with a higher score representing a higher level of loneliness. This measure has been used in large-scale population telephone surveys and has good reliability (Hughes et al., 2004). In our sample, the Cronbach’s alpha for the measure was 0.80.

#### Covariates

Covariates included in our models were chosen based on the literature (Plassman et al., 2010). The covariates initially considered for selection included demographics (age, sex, and race), socioeconomic status (years of education, marital status, living arrangement, and annual household income), health status (number of reported health conditions and depression), lifestyle factors (smoking and exercise), and an Alzheimer’s disease risk gene (APOE). The covariates retained in the final models were those that have demonstrated statistically significant associations with the outcome variable (i.e., cognitive function). These covariates included age (in years), education level (0 = “≤ 12 years”, 1 = “ > 12 years”), race (1 = white, 2 = black, 3 = other race), survey wave (time), household income (in quartiles), number of health conditions (range 0–8), and physical exercise (0 = no, 1 = yes). All of these variables were measured in HRS.

### Statistical analysis

Descriptive statistics were computed for sample characteristics. We conducted Little’s test for missing completely at random (MCAR) (Little, 1988) and found that data were missing completely at random in our sample. We implemented the expectation-maximization (EM) imputation to acquire robust estimates (Allison, 2009).

Both mediation and moderation effects of loneliness on the relationship between sensory impairment and cognitive decline were examined in the same model using the two-step longitudinal parallel-process (LPP) analysis (Hooke et al., 2018). In the first step, the intercept and slope of the longitudinal outcome, cognitive function, were estimated from multilevel modeling with SAS Proc Mixed. Age (centered at its mean of 81), as opposed to wave, was used as the time-varying random factor. We controlled “wave” to account for attrition represented by the status of a participant being measured or unmeasured for whatever the reason at the time of each wave. Thus, the intercept of cognitive function represents the average status of cognitive function at age 81. The slope represents the rate of change in cognitive function as people age by 1 year. By estimating the intercept and slope of cognitive function in the first step, LPP allows us to explore both cross-sectional and longitudinal relationships between sensory impairment, loneliness, and cognitive function in the second step. At the same time, the predicted values of sensory impairment and loneliness after controlling for the covariates were processed in the first step for the following SEM modeling. Since we used 1 wave of data for sensory impairment and loneliness, the predicted values of each of these variables in the first step were produced by using SAS Proc Logistic (for hearing and vision impairment) and Proc Genmod (for loneliness) with age as the predictor controlling for covariates.

In the second step, hearing impairment and visual impairment were modeled separately in structural equation modeling (SEM) to test the mediation and moderation effects of loneliness on the longitudinal relationship between sensory impairment and cognitive function using IBM SPSS Amos (Arbuckle, 2014). The moderation effects of loneliness on the longitudinal relationship between sensory impairment and cognitive function were examined by computing two interaction terms of visual impairment*loneliness and hearing impairment*loneliness in the SEM. For the final result, we used 1-tailed tests to test our directional hypotheses because prior literature consistently reported results in one direction (Lara et al., 2019; Shukla et al., 2020).

**FIGURE 2:**
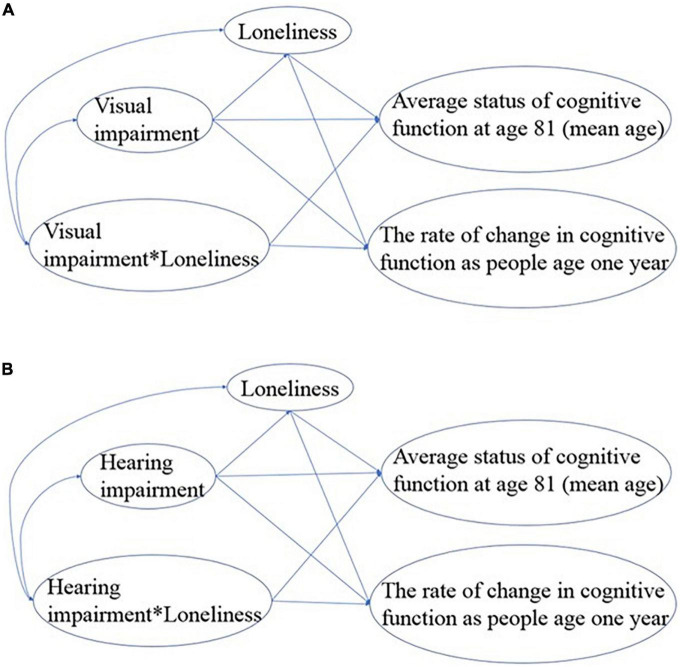
Initial mediation and moderation SEM models for **(A)** vision and **(B)** hearing impairment.

Specifically, we first fit a full SEM model for each modality of sensory impairment (Figure 2) based on our hypothesis. To achieve the most parsimonious model, we removed the non-significant paths one-by-one based on their *p*-values and interpretability. The model fit was evaluated using the following model-fit indices: Chi-square of the estimated model (χ^2^), goodness of fit index (GFI), normed fit index (NFI), incremental fit index (IFI), relative fit index (RFI), comparative fit index (CFI), and root mean square error of approximation (RMSEA). A non-significant Chi-square value (*p* > 0.05) suggests a good overall model fit to the data. For GFI, NFI, IFI, RFI, and CFI, values larger than 0.90 indicate that the model provides a good fit to the data, whereas the RMSEA value should be below.06. The fit indices and their criteria are commonly recommended in the literature (Hu and Bentler, 1999; Kline, 2015). Based on the general consensus that the number of participants needs to be 10 per estimated parameter (Schreiber et al., 2006), our analyses have a satisfactory sample size.

## Results

### Descriptive statistics

Characteristics of the sample are shown in Table 1. At baseline, in a total sample of 243 older adults, the average age was 81.08 ± 5.40. There were more females (53.5%) than males and more white participants (79.84%) than other races. For the modeled variables, the average cognitive score was 19.66 ± 5.47. The average level of loneliness was 1.53 ± 0.54. One hundred and twenty-three (50.62%) of the older adults did not have hearing impairment, but 101 (41.56%) of them had hearing impairment. As for visual impairment, 154 (63.37%) of the older adults did not have visual impairment, but 84 (34.57%) had visual impairment.

**TABLE 1:** Descriptive statistics of variables from HRS and ADAMS in this study at baseline (*N* = 243).

	*N* = 243 (%) or Mean ± SD	Range
Age	81.08 ± 5.40	73–98
**Gender**		
Male	113 (46.50)	
Female	130 (53.50)	
**Race**		
White	194 (79.84)	
Black	42 (17.28)	
Other	7 (2.88)	
Education		0–17
= 12 years	152 (62.55)	
>12 years	91 (37.45)	
**Annual household income (mean of each quartile)**		
Highest quartile	81,899.48 (48826.46)	40,380.00–260,016.00
Second quartile	32,041.28 (4925.56)	23,492.00–39,600.00
Third quartile	18,155.59 (3279.48)	12,960.00–23,232.00
Lowest quartile	9,486.82 (2405.18)	2,400.00–12,848.00
Health conditions	2.40 ± 1.43	0–6
**Physical exercise**		
Yes	58 (23.87)	
No	185 (76.13)	
Cognition	19.66 ± 5.47	2–32
Loneliness	1.53 ± 0.54	1.00–3.00
**Hearing impairment**		
No impairment	123 (50.62)	
Impairment	101 (41.56)	
Not assessed/Don’t know/Refused	19 (7.82)	
**Visual impairment**		
No impairment	154 (63.37)	
Impairment	84 (34.57)	
Not assessed/Don’t know/Refused	5 (2.05)	

SD, standard deviation.

### Mediation and moderation effects for visual impairment

The initial model fit indices for the SEM model (Figure 2) to examine the mediation and moderation effects of loneliness were not satisfactory (χ^2^(1) = 8.622, *p* = 0.003; GFI = 0.986, NFI = 0.994, IFI = 0.994, RFI = 0.936, CFI = 0.994; and RMSEA = 0.177), and indicated that the model needed further improvement. The estimated path coefficients were provided in Supplementary Figure 1.

**FIGURE 3:**
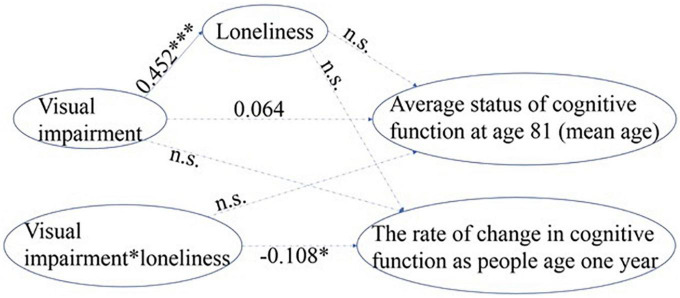
Standardized path coefficient estimates from the most parsimonious model for visual impairment. a. 1-tailed tests, *p < 0.05, ^***^p < 0.001. b. The error terms and correlational paths are omitted for clarity. c. The dotted paths with path coefficients shown were remained in the final model but were non-significant, the dotted paths without coefficients were deleted during the process of improving model fit (n.s.).

To obtain a parsimonious, best-fit model, the initial model was modified by removing non-significant paths based on statistical modification indices produced by IBM SPSS Amos as well as on theoretical interpretability. A final model was estimated as shown in Figure 3 in which the model fit indices for the final model improved and were all satisfactory (χ^2^(4) = 3.779, *p* = 0.437, GFI = 0.994, NFI = 0.997, IFI = 1.000, RFI = 0.993, CFI = 1.000, and RMSEA = 0.000).

Our results show that older adults who had visual impairment experienced more severe loneliness (standardized β = 0.452, *p* < 0.001). There was no significant association between loneliness and the rate of decline or the average status of cognitive function, suggesting no mediation effect of loneliness. The potential moderation effect of loneliness was represented by the interaction term of visual impairment*loneliness, which demonstrated a significant association with the rate of cognitive decline (standardized β =−0.108 [or-0.032, unstandardized], *p* < 0.05), suggesting a role of loneliness in moderating the effects of visual impairment on the rate of cognitive decline. In other words, for older adults who had visual impairment and felt lonely, their cognitive function declined by 0.032 more points per year than older adults who had visual impairment but did not feel lonely.

### Mediation and moderation effects for hearing impairment

The initial model fit indices for the SEM model (Figure 2) to examine the mediation and moderation effects of loneliness were not all satisfactory (χ^2^(1) = 7.898, *p* = 0.005; GFI = 0.987, NFI = 0.993, IFI = 0.994, RFI = 0.931, CFI = 0.994, and RMSEA = 0.169), and indicated that the model did not fit well. The estimated path coefficients were provided in Supplementary Figure 1.

**FIGURE 4:**
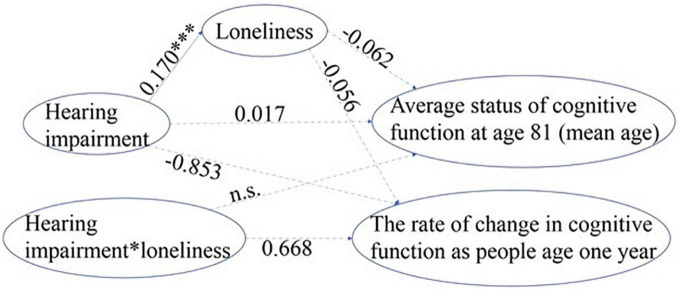
Standardized path coefficient estimates from the most parsimonious model for hearing impairment. a. 1-tailed tests, *p < 0.05, ^***^p < 0.001. b. The error terms and correlational paths are omitted for clarity. c. The dotted paths with path coefficients shown were remained in the final model but were non-significant, the dotted paths without coefficients were deleted during the process of improving model fit (n.s.).

As previously described for visual impairment, we took a similar approach to obtain a parsimonious, best-fit model for hearing impairment. The initial model was modified by removing non-significant paths. A final model was reached as shown in Figure 4 in which the model fit indices for the final model improved and were all satisfactory (χ^2^(1) = 0.172, *p* = 0.678, GFI = 1.000, NFI = 1.000, IFI = 1.001, RFI = 0.999, CFI = 1.000, and RMSEA = 0.000).

Loneliness did not mediate or moderate the relationship between hearing impairment and cognitive decline (Figure 4). Older adults with hearing impairment had more severe loneliness (standardized β = 0.170, *p* < 0.001) than those with normal hearing. However, the path coefficients from loneliness to both the rate of change in cognitive decline (slope) and the average status of cognitive function at age 81 (intercept, centered at mean age) were non-significant, indicating no mediation effect of loneliness on the association between hearing impairment and cognitive decline. The interaction term between hearing impairment and loneliness was also not significant for either the rate of decline in cognitive function or the average cognitive status at age 81, suggesting loneliness did not moderate the relationship between hearing impairment and cognitive decline.

## Discussion

Our study examined potential mediation and moderation effects of loneliness on the association between sensory impairment and cognitive decline in a national sample of older adults in the U.S. We found that loneliness moderated the association between visual impairment and the rate of decline in cognitive function, but we did not find a moderating effect of loneliness on the relationship between hearing impairment and the rate of cognitive decline. Even though we found that both hearing and vision impairment were significantly associated with increased severity of loneliness, we did not find a mediation effect of loneliness on the association between either hearing impairment or visual impairment and cognitive decline.

The potential detrimental impact of loneliness on health has been studied for decades (Cacioppo et al., 2002). In 2020, the implications of enforced social distancing during the COVID-19 pandemic has brought the adverse effects of both loneliness (i.e., perceived social isolation) and social isolation to the attention of both researchers and clinicians (Wu, 2020). The potential moderating role of loneliness on the associations among risk factors and health outcomes has been examined in other topic areas and populations such as stress and sleep among adolescents (Norman et al., 2011; Doane and Thurston, 2014; Zeligman et al., 2017), but little research has been done regarding the sensory-cognition associations. To our knowledge, only one recent study has explored the cross-sectional inter-relationships between sensory impairment, loneliness, and cognitive function using data from the Canadian Longitudinal Study of Aging (CLSA) (*N* = 30,029), but no significant moderating nor mediating effects of loneliness were found. Our study also did not find that loneliness moderated the cross-sectional associations between sensory impairment and average cognitive function (intercept, centered at mean age). However, we found loneliness moderated the longitudinal association between visual impairment and the rate of decline in cognitive function. Our findings imply that elevated levels of loneliness may exacerbate the negative impact of visual impairment on the rate of cognitive decline. Despite the increased challenges for social participation, older adults with visual impairment should try to stay engaged with family, friends, and community. For family caregivers and clinicians, our findings signal the importance of ameliorating loneliness among older adults, especially among those with visual impairment, by focusing on ensuring sufficient support and companionship.

Although we found loneliness moderated the association between visual impairment and cognitive decline, this moderating effect did not exist for hearing impairment. We suspect that this differentiated relationship is related to the different physical, social, and emotional challenges caused by vision and hearing impairment. Older adults with visual impairment may have even more limited physical mobility than those with hearing impairment. It might be easier for older adults with hearing impairment to adapt to the hearing-related challenges they face (Mick et al., 2018). For example, some physical and mental activities (e.g., walking for exercise, card games) may be still appropriate for older adults with hearing impairment but not for those with visual impairment. In addition, visual impairment may diminish older adults’ opportunities for outdoor activities and social interactions by limiting transportation options, which could increase the risk of feeling stressed or frustrated.

Our study found a significant association between both types of sensory impairment and elevated loneliness severity. Our finding is consistent with previous studies (Alma et al., 2011; Mick et al., 2018). Older adults with sensory impairment are likely to encounter difficulties in communication with families and friends. Therefore, sensory-impaired older adults’ social relationships can be harmed by miscommunications and misunderstandings (Crews and Campbell, 2004; Mick et al., 2014; Guthrie et al., 2016). Consequently, older adults with sensory impairment may feel negative emotions (e.g., frustration, anger, and stress) and tend to passively or intentionally withdraw from social interactions due to functional and communication difficulties (Mick et al., 2014, 2018).

The pathways connecting the association between sensory impairment and cognitive decline are largely unknown (Wayne and Johnsrude, 2015; Whitson et al., 2018). Sensory impairment has been hypothesized to cause cognitive decline through a pathway of loneliness (Lin et al., 2011a,b; Fulton et al., 2015). Our study is one of the few that has used longitudinal data to explore the potential mediating role of loneliness on the longitudinal associations between sensory impairment and cognitive decline (Maharani et al., 2019). However, we did not find a significant mediation effect of loneliness due to a lack of associations between loneliness and cognitive decline. This finding is contradictory to the findings from previous longitudinal studies (Donovan et al., 2017; Zhong et al., 2017; Maharani et al., 2019). A few factors may have contributed to this discrepancy. Previous studies that examined the longitudinal associations between loneliness and cognitive decline have primarily used memory to represent cognitive function (Donovan et al., 2017; Zhong et al., 2017) instead of using a multi-dimensional global cognition measure. These previous studies used a one-item measure of loneliness that may provide an unreliable estimate of loneliness (Donovan et al., 2017; Zhong et al., 2017). In contrast, we used the 3-item UCLA Loneliness Scale in our analysis, which has shown satisfactory reliability in our sample. However, while other studies used multiple waves of data of loneliness, we only had information on loneliness at baseline due to the study design of HRS (Smith et al., 2017). Nonetheless, our study is the first using data from the HRS and ADAMS to explore the role of loneliness in mediating the effect of objectively measured sensory impairment on cognitive decline.

Our study has some limitations. First, the measure of cognitive function in HRS was administered by a mixture of telephone and face-to-face interviews. Hearing function may have affected performance on the cognitive measure, especially for those that were conducted over the phone. Older adults with hearing impairment may also have a lower likelihood of completing the HRS TICS. However, the HRS team has found no measurable differences in cognitive performance based on the mode of the test administration (Herzog and Rodgers, 1999). Second, we did not have information about the time of onset of sensory impairment, which limits our understanding of how the length of time living with sensory impairment and adaptation to sensory impairment may influence its relationship to cognitive decline. Third, the study’s generalizability should be treated with caution. Although the ADAMS Wave A sample is a representative “snapshot” of the U.S. population (as of 2002), the data from ADAMS Wave C used in the present study included the participants who were (a) without a diagnosis of dementia at Wave A or B, (b) not deceased, and (c) able to complete the HRS TICS, thus they may be healthier than the general population. Fourth, vision and hearing status are subject to change as individuals age, yet we only used one time point of measured vision and hearing impairment because relatively few individuals had multiple sensory measurements due to the study design of ADAMS. Relatedly, due to this one-time measurement of both loneliness and the sensory impairment variables, we cannot establish temporal directions or causal relationships for the identified associations. Future studies should consider collecting longitudinal data for both sensory impairment and loneliness to provide stronger evidence for the plausible relationships.

## Conclusion

Our study examined the inter-relationships among sensory impairment, loneliness, and cognitive decline using longitudinal data. We used objectively measured sensory function. The measures of loneliness used in our study have also been widely used and have demonstrated satisfactory psychometric properties. Our findings suggest that vision and hearing impairment each has a different pattern of associations with cognitive decline, and loneliness may moderate the relationship between visual impairment and cognitive decline. Future studies that seek to understand the inter-relationships among sensory impairment, psychosocial factors, and cognitive decline using strong validated measures, larger sample sizes, and multiple waves of data in loneliness are warranted. Future studies should also consider examining if improving social connectedness would help slow down or even prevent future cognitive decline.

## Data availability statement

Publicly available datasets were analyzed in this study. This data can be found here: https://hrs.isr.umich.edu/data-products.

## Ethics statement

The studies involving human participants were reviewed and approved by the Duke University IRB. Written informed consent for participation was not required for this study in accordance with the national legislation and the institutional requirements.

## Author contributions

SG had primary responsibility for study conceptualization, data analysis, interpretation, and preparing the manuscript. EM, BW, WP, XD, and BP contributed to the study significantly by advising on the study design, reviewing the analysis results, revising the analytical strategies, and making crucial revisions to the manuscript. All authors contributed to the article and approved the submitted version.

## Supplementary material

Supplementary Figure 1 Initial mediation and moderation SEM full models and path estimates for **(A)** vision and **(B)** hearing impairment. a. 1-tailed tests, **p* < 0.05, ^***^*p* < 0.001. b. The error terms and correlational paths are omitted for clarity. c. The dotted paths were non-significant.
