# Supplementary figures and images for: Sensory impairment and cognitive decline among older adults: An analysis of mediation and moderation effects of loneliness
Source: Front Neurosci. 2023 Jan 9;16:1092297. doi: 10.3389/fnins.2022.1092297 (PMC9869267; doi:10.3389/fnins.2022.1092297)

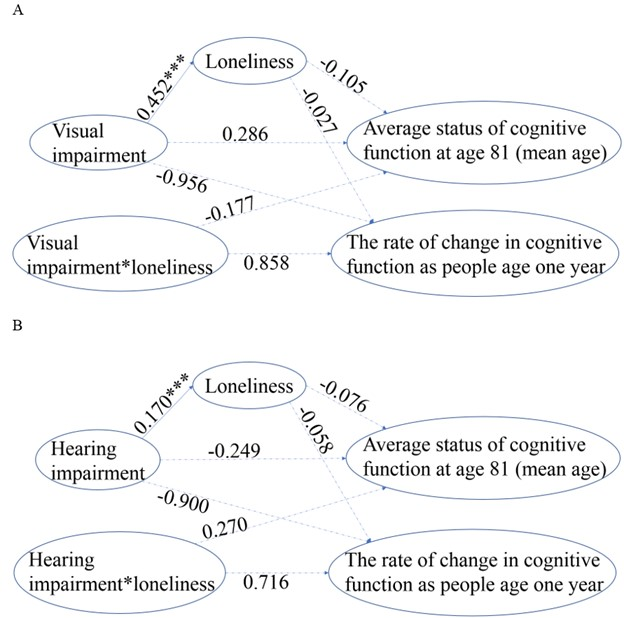

Supplement: Supplementary Figure 1 — Initial mediation and moderation SEM full models and path estimates for (A) vision and (B) hearing impairment. a. 1-tailed tests, *p < 0.05, ***p < 0.001. b. The error terms and correlational paths are omitted for clarity. c. The dotted paths were non-significant. [file Image_1.TIFF]
